# Genetic Diagnostics and Phenotypic Profiling of a Girl With Autosomal Recessive Intellectual Developmental Disorder and Autism

**DOI:** 10.7759/cureus.74379

**Published:** 2024-11-25

**Authors:** Silvia Lakatosova, Gabriela Repiska, Alica Valachova, Barbara Raskova, Ivan Belica, Lukas Patrovic, Daniela Ostatnikova, Michal Konecny

**Affiliations:** 1 Genetics, Institute of Physiology, Academic Center for Autism Research, Faculty of Medicine, Comenius University in Bratislava, Bratislava, SVK; 2 Medical Genetics, Faculty Hospital Trenčín, Trenčín, SVK; 3 Psychology, Institute of Physiology, Academic Center for Autism Research, Faculty of Medicine, Comenius University in Bratislava, Bratislava, SVK; 4 Radiology, Jessenius Diagnostic Center, Nitra, SVK; 5 Laboratory of Genomic Medicine, GHC Genetics SK Ltd. Science Park, Comenius University in Bratislava, Bratislava, SVK; 6 Biology, Institute of Biology and Biotechnology, Faculty of Natural Sciences, University of St. Cyril and Methodius in Trnava, Bratislava, SVK

**Keywords:** adaptive functioning, autism, genetic diagnostics, intellectual disability, whole exome sequencing

## Abstract

In this article, we present a case study of a five-year-old girl with autism and developmental delay, conducted at the Academic Center for Autism Research in Bratislava, Slovakia. The girl was diagnosed using Autism Diagnostic Observation Schedule-Second Edition (ADOS-2) and Autism Diagnostic Interview-Revised (ADI-R) instruments and met the criteria for autism spectrum disorder. Intellectual functioning was in the markedly below-average range, as indicated by the Snijders-Oomen Nonverbal Intelligence Test-Revised (SON-R) examination, and her level of adaptive functioning was significantly reduced. Neurological signs included atypical leukoencephalopathy, hypotonia, sensorineural hearing loss, and sleep disturbances. The patient underwent genetic testing, including microarray-based copy number variation (CNV) detection, which yielded negative results. However, whole exome sequencing (WES) analysis pointed out a damaging homozygous variant in the EIF3F (Eukaryotic Translation Initiation Factor 3 Subunit F) gene, confirming the diagnosis of intellectual developmental disorder autosomal recessive 67. Segregation analysis in the family revealed that the asymptomatic parents were carriers of the pathogenic variant in EIF3F. Our study contributes to the phenotypic profiling of this rare syndromic neurodevelopmental disorder and points out the irreplaceability of WES analysis in genetic diagnostics of autism and developmental delay. This appeals to the need for WES to be accepted as a routine diagnostic tool in Slovakia.

## Introduction

Autism spectrum disorder (ASD) is a neurodevelopmental disorder with a complex etiology. The genetic architecture of ASD includes rare damaging variants in autism candidate genes, as well as numerous common variants in genes associated with autism determined by genome-wide association studies [1]. This genetic complexity and the possible polygenic effect make the genetic diagnostics of ASD challenging. In approximately 25% of cases, the genetic cause is successfully determined. These include syndromic autism, where autism is part of a well-defined genetic syndrome, or molecularly defined autism, where DNA variants or copy number variants (CNVs) are identified using next-generation sequencing (NGS) or microarray-based methods [2]. Intellectual disability represents a frequent comorbidity in ASD [3,4]. Some patients with intellectual disability also exhibit autism or autistic traits.

In this article, we present a phenotypic description of a girl diagnosed with an autosomal recessive intellectual developmental disorder and ASD, caused by a homozygous missense variant c.694T>G, p.(Phe232Val) in the EIF3F (Eukaryotic Translation Initiation Factor 3 Subunit F) gene. The genetic diagnostic procedure that led to the capture of this causal variant was the whole exome sequencing (WES) approach. This case study was conducted at the Academic Center for Autism Research (ACAR), a research center based at the Institute of Physiology, Faculty of Medicine, Comenius University in Bratislava. Patients diagnosed at ACAR agree to participate in research projects. The mother of the child who is a subject of this case study provided informed consent for participation in the research, and the study was part of an autism research project approved by the Ethics Committee of the Faculty of Medicine, Comenius University in Bratislava and Bratislava University Hospital, under approval number 75/2021.

## Case presentation

A female patient was examined at the ACAR in Bratislava and the Department of Medical Genetics, Faculty Hospital in Trenčín, at the age of five years and eight months. The girl was from the second pregnancy, which had no complications. The labor occurred in the 41st gestational week spontaneously, with a birth weight of 3660 g, height of 51 cm, and an Apgar score of 10/10, without newborn icterus. Her early psychomotor development was delayed; she began crawling at 12 months and walked independently at 23 months. At the time of examination, she was eutrophic, proportional, with normocephaly (head circumference 51 cm, in the 25th-50th percentile), and had thinner hair. The patient had minor facial signs, including a higher forehead, hypotelorism, convergent strabismus, a prominent nasal root, a shorter philtrum, microretrognathia, a prominent maxilla, and slightly atypical weaker-shaped ears with smaller lobes. No other somatic abnormalities were noted. Her gait was broader based with flexed forearms. The girl showed stereotypic hand-shaking movements while expressing joy. Her vocabulary was restricted to 10 words, and she had urinary and stool incontinence.

The maternal grandfather had been diagnosed with depression, and her maternal aunt and uncle had schizophrenia. She has an older sister with no medical conditions. The patient is under the care of several clinical specialists, including neurologists, otorhinolaryngologists, psychologists, and a speech therapist. She was diagnosed with a bilateral mild sensorineural hearing defect, pervasive developmental disorder, delayed psychomotor development, and intellectual disability.

Neurological assessment

**Figure 1:**
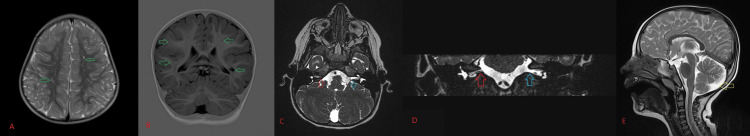
MRI scan of the patient at the age of 24 months (A, B) Cystic enlargement of the Virchow-Robin perivascular spaces (green arrows; A: axial T2-weighted sequence, and B: T1-weighted turbo inversion recovery sequence). (C, D) Right internal auditory canal stenosis (red arrow; T2-weighted isotropic sequence, axial and coronal planes) compared to a normal left internal auditory canal (blue arrow; T2-weighted isotropic sequence, axial and coronal planes). (E) Cisterna magna permagna (yellow arrow; T2-weighted sequence, sagittal plane).

The patient has a global developmental delay, impaired intellectual development, impaired speech development, hypotonia, and sleep disturbances. An MRI scan at 24 months of age showed randomly distributed cystic dilatations of the Virchow-Robin spaces bilaterally in the white matter, with a maximum concentration in the left parietal-temporal-occipital site, indicating atypical leukoencephalopathy. The findings also included an asymmetrically narrowed right internal auditory canal and cisterna magna (Figure 1).

The Children's Sleep Habits Questionnaire (CSHQ) was used to assess sleep problems in the proband [5]. The patient overreached the cut-off score (48, with a cut-off of 41), indicating the presence of sleep disturbances, with bedtime resistance and night waking being the most pronounced issues.

Psychological assessment

The Autism Diagnostic Interview-Revised (ADI-R) and the Autism Diagnostic Observation Schedule-2 (ADOS-2) instruments were used both as diagnostic tools and to quantify the severity of autism symptoms [6,7]. The results of both examinations indicated the presence of typical ASD manifestations, with moderate severity of symptoms, including impaired speech and intellect.

The SON-R 2 ½-7, a nonverbal measure of general intelligence, was used to measure the level of developmental delay [8]. The patient's intellectual performance was in the markedly below-average range at the time of the assessment. However, due to the child's lack of cooperation, these results were only orientational, as only the Puzzles, Categories, and Analogies subtests were successfully administered. The ability to use mental images to solve problems, including mentally rotating objects, interpreting how objects change when moving in space, perceiving and manipulating spatial configurations, and maintaining spatial orientation, was significantly in the below-average range. The proband’s performance in fluid intelligence tasks, which use analytical-synthetic thinking and problem-solving abilities, was also in the significantly below-average range. The proband's intellectual abilities were orientationally at a level typical for children aged two years and two months.

**Figure 2:**
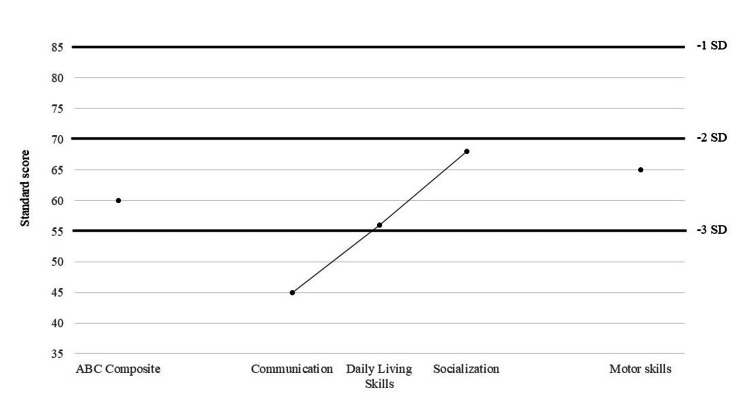
Adaptive behavior profile of a patient at the age of five years Adaptive behavior profile (composite score and domains) and motor skills: mean = 100, SD = 15. ABC composite: Adaptive behavior composite, a summary score that represents a composite measure of adaptive behavior.

The Vineland Adaptive Behavior Scales-3 [9] questionnaire was used to measure the level of daily independent functioning, known as adaptive behavior. Adaptive behavior is defined as the collection of conceptual, social, and practical skills learned by people to enable them to function in everyday life. The communication domain assesses the individual's ability to communicate both verbally and nonverbally, including expressive, receptive, and written communication. The daily living skills domain covers personal (e.g., dressing and hygiene), domestic (e.g., household chores), and community (e.g., managing money) activities. The socialization domain covers interpersonal relationships, play and leisure activities, and coping skills. The results showed a deeply below-average adaptive behavior profile in all subdomains of the test, including communication, daily living skills, socialization, and motor skills, as well as an overall adaptive behavior score (Figure 2).

The Behavior Rating Inventory of Executive Function - Preschool Version (BRIEF-P) was used to assess executive dysfunction in the proband [10]. According to the BRIEF-P ratings by her parents, the proband has significant difficulties with executive functions, with clinically elevated scores on the Inhibit, Working Memory, and Plan/Organize scales as well as the Inhibit Self-Control Index (ISCI) and the Emergent Metacognition Index (EMI). Her score on the emotional control scale was also mildly elevated, although it did not reach clinical significance, suggesting difficulties with emotional reactions to seemingly minor problems. Poor behavior regulation may manifest as high levels of physical activity or a tendency to interrupt and disrupt group activities. Due to deficits in attention, working memory, and inhibitory self-control, the proband has problems working in a systematic and organized manner. The results also revealed deficits in metacognitive skills, indicating a poor ability to use information from working memory to guide her performance or behavior.

Problem behavior was assessed using three questionnaires: the Behavior Problems Inventory (BPI-01), the Children's Scale for Hostility and Aggression: Reactive/Proactive (C-SHARP), and the Nisonger Child Behavior Rating Form (NCBRF) [11]. The first two instruments did not reveal any behavioral abnormalities. The patient's score for overly sensitive behavior on the NCBRF was at the 80th percentile of the normative sample for the same age category and collapsed gender, meaning that 80% of the normative sample population showed a lower level of sensitive behavior, including emotional sensitivity and responsiveness, such as being easily upset, having strong emotional reactions to events, and showing sensitivity to criticism or changes in routine.

Genetic assessment

Cytogenetic examination showed a normal karyotype of 46, XX. Microarray analysis of CNVs (aCGH) showed negative findings. DNA methylation analysis did not reveal a deletion or imprinting defect of 15q11.2-q13. WES, using a virtual panel for autism and intellectual disability based on human phenotype ontology (HPO) terms and the Simons Foundation Autism Research Initiative (SFARI) database, pointed out several suspected variants, including a homozygous missense variant c.694T>G, p.(Phe232Val) in the EIF3F gene with potentially pathogenic predictions and several variants of uncertain clinical significance (Table 1).

**Table 1:** List of variants derived from the WES analysis using a virtual panel for autism and intellectual disability dbSNP ID: Single nucleotide polymorphism identification in database dbSNP; HOM: Homozygote; HET: Heterozygote; AR: Autosomal recessive; AD: Autosomal dominant.

Gene	Reference sequence	cDNA	Protein	Zygosity	dbSNP ID	Pathogenicity (Varsome)	Inheritance (OMIM)
EIF3F	NM_003754	c.694T>G	p.(Phe232Val)	HOM	rs141976414	4	AR
NF1	NM_001042492	c.2065G>A	p.(Val689Met)	HET	rs771784652	3	AD
NRXN2	NM_015080	c.157C>T	p.(Leu53Phe)	HET	rs568060819	3	-
PCDHA3	NM_018906	c.503T>C	p.(Leu168Ser)	HET	-	3	-
PRODH	NM_001195226	c.229_230delinsCA	p.(Trp77Gln)	HET	rs386819653	3	AD, AR
SHANK2	NM_012309	c.3788C>T	p.(Thr1263Met)	HET	rs1179142044	3	-
ZNF517	NM_001317936	c.1023_1025delinsTGC	p.(Val342Ala)	HET	-	3	-

The EIF3F gene is associated with autosomal recessive inheritance of a defined disease entity (OMIM: PS249500). Since the patient is a homozygote for potentially pathogenic variant c.694T>G, a segregation analysis of the variant with the disorder was conducted in the patient's family members. Out of all family members, only the parents' samples were available. The segregation analysis revealed that both asymptomatic parents were heterozygous carriers of c.694T>G, confirming its causality and the diagnosis of intellectual developmental disorders autosomal recessive 67. WES analysis also showed the presence of six other variants of uncertain clinical significance in genes related to autism and/or intellectual disability. A missense variant c.2065G>A, p.(Val689Met) in NF1 was a subject of segregation analysis due to its autosomal dominant pattern of inheritance; however, its clinical significance was not confirmed due to its presence in the asymptomatic father. The identification of the causal variant c.694T>G, p.(Phe232Val) in the EIF3F gene indicates a 25% recurrence risk in potential siblings of the patient, due to its autosomal recessive mode of inheritance, as explained by the clinical geneticist to the parents of the proband.

## Discussion

Intellectual developmental disorder, autosomal recessive 67, belongs to the phenotypic series of autosomal recessive intellectual developmental disorders (OMIM: PS249500), comprising 70 diagnostic categories connected to various monogenic alterations with common features of intellectual disability and other accompanying symptoms. In particular, intellectual developmental disorder autosomal recessive 67 is caused by a homozygous mutation in the EIF3F gene located in the 11p15.4 region. This gene was first identified and functionally validated in 2018 [12]; however, the population frequency of this syndromic disorder has not been determined since then. The EIF3F gene encodes eukaryotic translation initiation factor 3 subunit F, which is a part of the eukaryotic translation initiation factor 3 complex. It contributes to translation initiation factor activity. The complex is capable of the RNA-protein and protein-protein interactions required to stimulate the translation initiation reactions dependent on eIF3.

According to the OMIM database, phenotypic features of the disorder caused by a defect in the EIF3F gene include global developmental delay, mildly to profoundly impaired intellectual development, speech delay or absence, hypotonia, variable facial dysmorphism, and, in some patients, sensorineural hearing defects, microcephaly, sleep disturbances, and autism spectrum disorder [13]. Several clinically significant variants have been previously described in EIF3F. The Varsome database reports two pathogenic variants: one missense and one frameshift variant [14]. Literature sources reporting EIF3F-related syndromic neurodevelopmental disorders are limited. A genome-wide contribution of recessive coding variation to developmental disorders was estimated by Martin et al. [12], who first time identified variant c.694T>G of the EIF3F gene. GnomAD database reports the frequency of a minor allele of the variant to be 0.12% among non-Finnish Europeans. The phenylalanine at position 232 is evolutionarily conserved and is predicted to stabilize the protein. The variant reduces the translation rate and proliferation rate. Martin et al. [12] found a homozygous c.694T>G/ p.(Phe232Val) variant in the EIF3F gene in nine patients with developmental disorders. All the patients had an intellectual disability, and some of them had seizures, behavioral difficulties, and sensorineural hearing loss. Hüffmeier et al. [15] reported the phenotypic description of 21 patients homozygous and one compound heterozygous for c.694T>G in the EIF3F gene. All affected individuals had developmental delays, including delayed speech development, and some patients also had behavioral problems, altered muscular tone, hearing loss, short stature, microcephaly, minor facial features, reduced sensitivity to pain, and gastrointestinal and ophthalmological symptoms. The patient in our study displays the majority of the phenotypic symptoms observed by these studies including developmental and speech delay, intellectual disability, hypotonia, sensorineural hearing loss, minor facial features, and autism spectrum disorders with medium severity scores. Psychological assessment of our patient brings detailed information on the level of adaptive functioning, executive dysfunction, and overly sensitive behavior.

Moreover, the MRI scan of our patient showed the signs of leukoencephalopathy, a disorder of brain white matter. Another case study engaging MRI revealed extensive supratentorial leukodystrophy; however, the patient was a compound heterozygote in EIF3F [16]. The precise mechanism of how the absence of EIF3F contributes to the phenotype manifestations remains unclear. The role of this translation initiation factor was described in skeletal muscles in maintaining their size and preventing muscle atrophy; in particular, EIF3F overexpression resulted in hypertrophy through modulation of protein synthesis via the mTORC1 pathway [17]. The role of decreased EIF3F expression in tumorigenesis has also been reported [18]. EIF3F was shown to be overexpressed in white matter compared to gray matter in various brain regions [19,20], suggesting its importance in myelination and white matter homeostasis, including regulation of oligodendrocyte functions. In addition, EIF3F was described to selectively regulate the translation of spinocerebellar ataxia type 8 RAN proteins that form aggregates in white matter regions that show demyelination and axonal degeneration [19], pointing out the importance of EIF3F in white matter homeostasis. White matter has a unique neurobiology and undergoes dynamic prolonged development with possible alterations in neurodevelopmental disorders including autism spectrum disorders.

Routine genetic diagnostic procedures in patients with neurodevelopmental disorders in Slovakia include array-based methods for the detection of CNVs, which are relatively common causal findings in this group of patients. However, single nucleotide variants or small insertions/deletions would be left unrecognized. Whole exome or genome sequencing procedures should be included as a part of genetic diagnostics in these patients to increase the chance of capturing causal or suspected DNA variants, as was the case in this presented study.

## Conclusions

Our case study contributes to the phenotypic description of a rare EIF3F-related syndromic neurodevelopmental disorder. The patient manifested with intellectual disability, autism, below-average adaptive functioning, executive dysfunction, and overly sensitive behavior. Neurological findings included leukoencephalopathy. The study further strengthens the importance of WES as an irreplaceable diagnostic tool for neurodevelopmental disorders including autism spectrum disorders. Although genetic diagnostics for neurodevelopmental disorders can be challenging due to polygenic architecture, noncoding variation, and incomplete penetrance, utilizing all available tools is essential to maximize the likelihood of identifying causative factors, which may help optimize management for patients and their families.
